# Phase I trials of antitumour agents: fundamental concepts

**DOI:** 10.3332/ecancer.2015.501

**Published:** 2015-01-19

**Authors:** Diego de Araujo Toloi, Denis Leonardo Fontes Jardim, Paulo Marcelo Gehm Hoff, Rachel Simões Pimenta Riechelmann

**Affiliations:** Cancer Institute of the State of São Paulo, São Paulo, Brazil; Faculty of Medicine, University of São Paulo, São Paulo, Brazil

**Keywords:** phase I study, oncology, molecular-targeted drugs, combination therapy

## Abstract

Phase I trials are an important step in the development of new drugs. Because of the advancing knowledge of cancer’s molecular biology, these trials offer an important platform for the development of new agents and also for patient treatment. Therefore, comprehension of their peculiar terminology and methodology are increasingly important. Our objectives were to review the fundamental concepts of phase I designs and to critically contextualise this type of study as a therapeutic option for patients with refractory cancer.

## Introduction

Phase I trials (a synonym of ‘dose-escalation study’ and ‘human pharmacology study’) are the first of the three stages of clinical trials and are classically designed to assess the safety and maximum tolerated dose (MTD) of new drugs. Studying pharmacokinetic and pharmacodynamic aspects of drug interactions is also a secondary objective of these trials. They are generally the first test of new agents on humans and are usually open-label, non-blinded studies on a small number of healthy and/or ill patients [1].

Thanks to the increase in the genetic knowledge of neoplasms, newer molecular targets are emerging as potential therapeutic alternatives. There are many drugs in development which aim to block canonical pathways associated with these alterations, which commonly occur only in a small segment of patients with cancer. In this context, phase I trials are a very good tool for monitoring treatment. They are so important that some regulatory agencies have considered results of phase I and II studies to be sufficient evidence to support and approve new drugs. However, in the vast majority of countries, including Brazil, approval also requires data from phase III trials [2].

In general, it is increasingly important for oncologists to understand the design and methodology of phase I trials because the number of these trials is highly likely to increase in the next few years. In addition to this, with the increase of multiplex molecular testing in oncology to identify druggable targets, these trials can present therapeutic alternatives for patients. Knowledge of phase I trials can also make it easier to refer candidate patients to specialist research centres.

There is currently a variety of methodologies for the execution of phase I trials, ranging from classic simple rule-based models, known as 3 + 3 models, to sophisticated computational models involving Bayesian algorithms. With the emergence of new targeted therapies, it has become evident that continuous modifications need to be made to the classic model of these trials for these new medicines. This is a way of increasing accuracy in dose finding and measuring their safety [3].

This article aims to discuss phase I trials, designs and objectives, as well as contextualising the subject in research and the oncological treatment of patients with advanced cancer.

## Context

**Figure 1.**
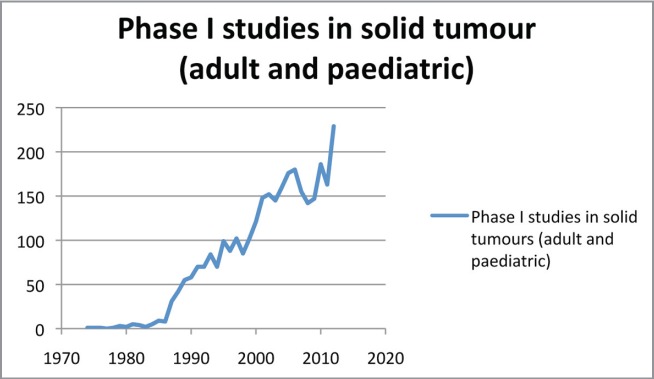
Phase I studies in solid tumours (adult and paediatric).

**Figure 2.**
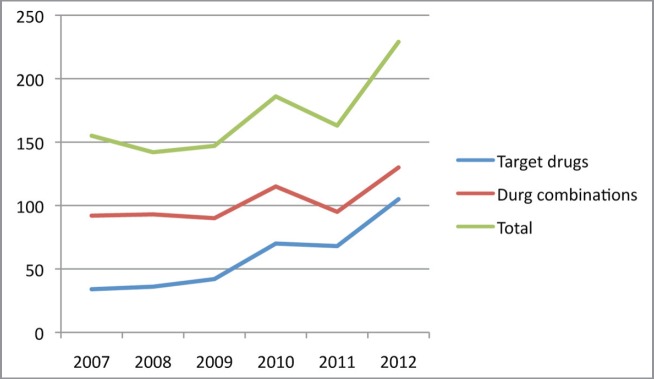
Phase I trials with target drugs and drug combinations in the past five years. [keys: target-drugs; combination; total].

We are currently seeing a growing number of new drugs in development. Data from the Pharmaceutical Research and Manufacturers of America (PhRMA) indicate that there are more than 800 oncological drugs in active clinical development, an increase of 143% in comparison with the previous decade (data from 2010) [3]. It is estimated that only one drug out of every 20 submitted to clinical trials in the area of oncology is commercialised. In addition, there has been a shift in investment in the pharmaceutical sector, with an estimated growth of nearly 55% [3]. Proof of this can be found in the significant increase of phase I trials in oncology. For example, when we search for the term ‘phase I’ on PubMed, 39,077 results appear; if we add the term ‘oncology’, the search engine finds 7396 articles, which corresponds to 18.93% of the publications associated with the term ‘phase I’ (search conducted in October 2014). A year-on-year analysis of phase I trials in solid, paediatric, and adult tumours clearly demonstrates an increasing trend in publications (Figures 1 and 2).

Phase I trials are an important step after the extensive performance of preclinical trials. These trials are an essential tool for interfacing between experimental laboratory knowledge and clinical application in patients. In oncology, they are generally offered to patients with incurable illnesses and in cases where there are no available effective therapies or promising studies in more advanced stages. The phase I study results are essential to the continuation (or not) of the next stages of development as phase II and III studies and later incorporating these results in clinical practice [3].

## Objectives of phase I trials

A few aspects should be highlighted before designing a phase I trial. It is important to ensure that preclinical trials of the medicine have been conducted appropriately. In this context, it is important to critically review the preclinical data in order to ensure the trustworthiness and accuracy of this information so it may serve as a hypothesis to be assessed in a phase I clinical trial. In the United States, submissions to the Investigational New Drug (IND) programme, part of the US Food and Drug Administration (FDA), contain information on the drug as well as data relating to the investigators, protocols, and nonclinical toxicological data [3]. It is important to note that ethics and quality in data collection, the use of good clinical practices, and quality standards are critical aspects of any trial—the reference document for Brazil is the ‘Document of the Americas’, which can be accessed on the website of the National Health Surveillance Agency (ANVISA) [4].

Phase I trials have their own concepts and terminology which should be understood for greater comprehension of their design and methodology (Table 1). The primary objective of phase I trials is to determine the MTD or the recommended phase II dose (RP2D) [5]. For cytotoxic agents, this dose shall be established through observation of unacceptable toxicity in a predetermined number of patients, based on the rationale of the efficacy–toxicity model. According to this traditional model, increasing doses correlate to greater response and, in turn, to greater toxicity; we are therefore getting an optimum level of the highest toxicity tolerated. However, the efficacy–toxicity curve for targeted drugs often features a plateau, meaning that increasing doses may not necessarily correlate with greater clinical benefit. The aim is to establish a dose which results in adequate target modulation and clinical activity. This may differ from the MTD, which makes the objective of studies of this type more complex. The pharmacokinetics and pharmacodynamics of the drug are among the parameters which may be established during phase I. In a phase I study, pharmacodynamics involves a broad range of studies of the possible effects that the drug produces on tumour tissue and healthy tissue. This may include aspects such as molecular studies inhibiting a certain intracellular target (when this is known), or assessing indirect markers which can express the drug’s biological activity, including alterations in imaging tests and even in clinical examinations [3]. The notion of the MTD is important even when the objective is to conduct pharmacodynamic assessments regarding whether or not the drug inhibits the predetermined target [6].

Alternatively, biologically effective dose (BED) is a term used for dose finding in molecular-targeted therapies. In this case, the variables involved in determining the BED would be the minimum level of serum concentration which can inhibit the molecular target, the inhibition percentage of the target marker, or the minimum level of expression of the molecular target (specific biological response) [7]. In practice, few studies are using these new concepts; however, such a change should become a trend in the next few years [8].

As secondary objectives, some phase I trials include correlation analyses which allow collection of preliminary information on the drug’s efficacy, which is particularly interesting for therapies such as molecular-targeted drugs, vaccines, and immunotherapies in which the efficacy–toxicity relationship is not linear as that of cytotoxic therapies. The early exploration and introduction of biomarkers for patient selection is quite important for molecular-targeted drugs because the population with the greatest probability of benefiting from a drug are selected early, costs are optimised, and the chances of success in the development of this drug are increased. In addition, phase I trials are currently the first test of the concept of new drugs. Putting it simply, it is established at this stage whether the drug is capable of doing what it was designed to do. If the drug does not show signs of clinical activity and is not capable of modulating its target, it is highly likely that its development will be abandoned.

Considering these objectives, a unicentric strategy should be considered when designing these studies, which differs from the design of phase II and III trials, which are often multicentric. In the case of phase I trials, there is a concern regarding the spread of patients in various centres because this implies different investigators caring for a limited number of patients, which may jeopardise an accurate evaluation of the drug’s toxicity profile [5, 9].

## Designs of phase I trials

Designs of phase I trials are usually divided between rule-based and model-based dose escalation methods. Escalation in rule-based methods is determined according to toxicity: the ‘3 + 3’ design, in which cohorts of three patients receive increasing doses of the drug (an example is the Fibonacci model of dose escalation); accelerated titration design, which takes into account the toxicity of each dose according to specific rules which allow faster dose increments (the dose-toxicity curve is updated for each cohort of patients for the programming of each inclusion stage) [5] pharmacologically-guided dose escalation (PGDE); and others such as the rolling six design, which allows between two and six patients per level (common in paediatric studies which aim to optimise the trial’s duration time in this age range) [10]. Model-based methods define the escalation pattern with the help of complex statistical methods such as the Bayesian method. Some model-based trial designs are: continual reassessment, escalation with overdose control (EWOC), and time-to-event models, which allow the inclusion of late-onset toxicities and include a therapy’s efficacy [11]. It should be remembered that model-based trials demand specialised statistical support, which increases the costs and complexity of the trials. Table 2 summarises the main phase I trial designs in oncology.

The advantages and disadvantages of rule-based and model-based designs are analysed when choosing the most appropriate design. Information relating to the drug and the availability of resources for continual assessment of pharmacokinetics and biostatistics should be considered, as well as the pros and cons in relation to the interpretation of data such as pharmacokinetics, variability between patients, and the determination of the RP2D [11]. The main objective of the designs is to reduce the number of patients receiving non-therapeutic doses without exposing them to severe toxicity. However, in practice, new designs do not always reduce the number of participating patients or the time to complete the trial [3]. For example, the 3 + 3 model can offer a lower risk of toxicity because of the slow escalation (from three in three patients), but also allows patients from the first cohorts to be exposed to suboptimal doses of the drug. The accelerated titration model does the opposite and treats the patient with doses approaching the MTD but entails a greater risk of adverse events. Knowledge of the different types of trials helps us to choose the most appropriate method for the scenarios in which the drug will be trialled (Table 2).

A review of 53 phase I trials following the continual reassessment design demonstrated that model-based designs can be safe, with toxicity in line with clinical expectations, meaning that these designs allow the observation of a shorter duration time and the assessment of multiple objectives (for example, the establishment of the MTD for patient subgroups). This review highlights the questions on the assessment of biomarkers in targeted drugs trials and expansion cohort studies, which shall be detailed later [12].

Regarding biologically effective dose finding, in addition to toxicity, model-based designs incorporate the bivariate continual reassessment method (CRM)—bCRM, and the trivariate CRM—tCRM, which is a measure of efficacy. In the first example of the design (bCRM), toxicity, and the measure of efficacy are considered binary parameters, and with a bivariate analysis four results are possible: no dose-limiting toxicity (MTD) and no efficacy, no MTD and presence of efficacy, presence of MTD without efficacy, and presence of both efficacy and MTD. In the second example (tCRM), there is an expected order of events which allows three possible sequential results for analysis: no efficacy and no MTD, efficacy without MTD, and severe MTD (making efficacy irrelevant). Appropriate algorithms are used in both examples which allow the establishment of the biologically-effective dose [7].

Phase I trials can present expansion cohorts, which can be one or more cohorts of patients which will be included after safe dose finding (generally RP2D). We are currently observing an increase in the use of these cohorts (12% of phase I trials on monotherapy in 2006 and 38% in 2011), mainly for non-cytotoxic drugs and in a multicentric scenario, with a median number of 17 patients included [13]. Assessing safety and efficacy is highlighted as one of the main objectives of these expansions, which allow the knowledge of new relevant toxicities and even the modification of the RP2D in some cases. An increase in the use of cohort expansions is expected, as the description of methods is important—for example, the number of patients to be recruited—as well as the definition of the objectives and parameters to be assessed to allow for better use of these data. It is important to note that although expansion cohorts are useful in helping to decide whether or not to monitor trials of a determined medication, they are not equivalent to phase II trials and should not substitute them [13].

The participating population is an important aspect to consider in the methodology of phase I trials. Patients invariably have advanced cancer which does not respond to other treatments. The eligibility criteria for phase I trials are also quite particular and commonly include at least three months’ life expectancy, performance status 0 to 1, absence of comorbidities, restriction in the use of other medicines, and preserved organic functions, although the patient has commonly undergone different treatments in the past. Inclusion of the enriched population, meaning selecting patients with a specific molecular profile, is becoming frequent. Treatment time is generally short (because of the progression of the disease) and laboratory and radiological assessments are frequently required.

Studies on the selection of patients have also assessed the potential risk of toxicity related to the application of a treatment cycle with the creation of nomograms composed of variables such as performance, laboratory examinations (creatinine, albumin, and aspartate transaminase clearance), type and quantity of drugs [14]. An appropriate selection of patients, in line with appropriately-chosen objectives and designs, is more likely to lead to a well-conducted trial, meaning that the results obtained are more reliable.

## Molecular-targeted agents and phase I trials

The formulation of phase I trials with molecular-targeted drugs is very complex because the linear relationship between dose and efficacy, observed with chemotherapeutic drugs, does not always occur in these cases. The objective of phase I trials with molecular agents is also different from those conducted with chemotherapeutic drugs (for example, in the determination of the lowest dose necessary to inhibit a molecular target) [3].

For the majority of molecular-target agents, the dose escalation stage is not conducted with response assessment at each dose level, as it may be through serial tissue assessments, as toxicity is the parameter usually monitored to determine the MTD. With this strategy, the MTD selected for later studies can exceed the minimum effective dose, which would be needed in order to obtain clinical benefits [15].

Studies also demonstrate that a significant percentage of dose-limiting toxicity of molecular-targeted agents occur after the first-cycle window, which is outside of the chosen period for dose finding. A review with 445 participating patients from a total of 36 phase I trials with molecular-targeted drugs demonstrated a total of 790 cases of toxicity during the first cycle and 1819 events afterwards. A total of 50% of patients demonstrated a greater intensity of toxicity after the first cycle. This review signals the importance of assessing late-onset toxicity during the determination of the recommended phase II dose (RP2D) in agents for chronic administration, like many molecular-target drugs [16]. It is important to consider the late toxicity profile and, later, the assessment in conjunction with an appropriate report on toxicity of major trials in order to define the final recommended dose and guidance on handling adverse events.

The interest in assessing a large number of molecular-targeted drugs resulted in the idea of phase zero trials. These are small trials on humans which test the specific effect on molecular targets before beginning the phase I trial proper. The idea of studying efficacy against targets through pharmacodynamic tests in an initial stage of the development of a molecular-targeted drug can help in establishing a dose for the phase I trial. One negative aspect of this approach is: using the result of a study with only some molecular targets and on a limited and small number of patients is questionable, given the knowledge of the complexity of the altered molecular pathways in the majority of neoplasms, because in reality it is only an exploratory premise. Another aspect which should be further discussed and looked into is the establishment of biological and statistical criteria which define the pharmacodynamic response and the EBD [5]. In addition, there is an ethical question regarding phase zero trials: the short period of time in which the drug is administered may not offer any benefit to the patient but may still cause adverse events.

## Phase I studies with drug combinations

In phase I trials, combinations occur with drugs that inhibit resistance systems, drugs with multiple targets, or whose interaction enables easier identification of toxicity profile and dose optimisation. The combination studies may be of single leg, or preventing different dosages from being evaluated [3].

For the preparation of a phase I study involving the combination of drugs, some aspects are important and must be considered: the presence of preclinical rationale, the combination should be theoretically superior to single agents, prior evidence of additive or synergistic effect (e.g., xenographic models), and knowledge on the combination’s toxicology, to look for the possibility of overlapping toxicities [3].

By studying the drug combination, the study’s objective shall be to find a combination of doses that have an acceptable rate of toxicity. In this case, the assumption of a direct relationship between dose and toxicity is not appropriate because it depends not only on the probability of individual toxicity, but also on the various possibilities offered by drug combinations [11]. For example, imagine two drugs administered in two ways and we assume that in the second modus there is increase in toxicity in one of the drugs and decrease in the other: to be able to forecast the order of toxicities by way of administration becomes uncertain [17].

Some design combinations choose a fixed dose of one drug and allow the dose of another to vary—in practice, it limits the review of the use of varying doses of the two drugs together, and presents significant risk of error if the initial fixed dose is not the most appropriate. The choice to maintain a fixed low dose and scale the other until DMT is established, and then perform the inverse is also done, and it is called the combined DMT final dose. However, these designs end up having little statistical power. Designs establishing toxicity probabilities in small number of combinations associated with a continuous assessment methodology seem to be promising models [17].

## Myths in relation to the phase I study

There are some misconceptions among the oncology communities not directly involved in the phase I studies but who often interfere with the referral and recruitment of patients. First is the notion that such studies are toxic and dangerous to patients. In a meta-analysis of 11,935 patients, the mortality related to participation in phase I was only 0.5% [18]. This figure is lower to the mortality described in subsequent phase II and phase III studies exploiting the same drugs [19]. There is also the idea that the tested drugs are generally ineffective and response chances are very low, especially for patients enrolled in the early cohorts with lower drug doses. The historical response rate for phase I studies is 6% [18], reaching 15% if the combination involves a chemotherapy. Interestingly, recent studies have shown that patients receiving lower doses of targeted therapies do not appear to derive less benefit than other patients included in later cohorts, [21] such as in the case of abiraterone [22]. Finally, it is not true that the concept of phase I studies are for terminal patients. In fact, most studies demand the inclusion of patients who have exhausted therapies that have proven benefit in overall survival (often in at least three months). Unfortunately, for the majority of metastatic solid tumours such benefit is inexistent beyond the second or third line of therapy. Furthermore, such studies are not only restricted for patients with excellent prognosis, but if there is adequate organ function and life expectancy of at least three months, then those patients can always be a candidate for a phase I study.

On the eligibility of participants, there is reservation as to the participation of the elderly in these studies. A recent presentation based on a review of 296 patients from 20 phase I studies which compared a subgroup aged less than 65 years and one aged over 65 years. There was no significant difference in terms of survival, and there was no correlation with the incidence of toxicities grade 3 and 4 [23]. Another recent publication with 5,401 participants from 162 studies, with 27% between 60 and 69 years of age, and 16% between 70 and 79 years age, showed that with increasing age and worsening performance there is increased likelihood of dose-limiting toxicity. However, with the risk deemed acceptable, it does not justify the restriction of elderly patients for these studies [24]. Thus, it is also necessary to promote the inclusion and participation of elderly patients in phase I studies because of the prevalence of this subgroup in epidemiological terms. It is also because of the probable non-commitment to the correct conduct and conclusion of these studies.

In the context of application to patients in which the standard therapy is exhausted, it is an important consideration to give proper clarification for the free consent of participants in phase I. To explain the objectives and risks, differentiating the concept of clinical study and standard treatment can mitigate a false concept of therapeutic intent, ensuring the conduct is in an appropriate and ethical manner. Aspects commonly pointed to this distinction include guidance on the scientific purpose, procedures, risks and uncertain benefits, alignment to conduct protocols and the objective of safety; also, in some cases, the therapeutic efficacy [25].

## Discussion

There is currently no consensus on the most appropriate design for phase I studies, requiring the review of the advantages and limitations of each design.

With the emergence of targeted therapies, the need to tailor the designs to the class of drugs as well as in relation to the use of biomarkers, e.g., patient selection and in drug combination studies, has become apparent. An expression increasingly commonly used when studying phase I studies is to ‘fail early and fail fast’, which expresses the goal of the new design models to dismiss a drug quickly if it fails to act against certain cancers, with exposure to fewer patients. Other therapeutic strategies involving radiotherapy, drugs with nuclear medicine and immunobiology, although not part of these papers, also deserve a special and individualised approach.

Studies comparing various design models can more objectively compare advantages and disadvantages in an attempt to establish the best indications for each different class of drugs. Similarly, they enhance the understanding of the molecular pathways of the disease and the variety of histologic tumour types should also assist with validation of biomarkers that can guide the choice and performance of such studies.

The ethics of phase I studies deserve to be remembered, especially during main challenges like the issue of risk–benefit, and the informed consent of participants. The concern to avoid toxicity and the use of suboptimal doses is present in study designs. This care, combined with the growing individualisation in the inclusions of patients in order to provide studies most likely to produce benefit, requires a cost–benefit review of the concept for this group of cancer patients. One possibility for this improved assessment can be the analysis of the impact on the quality of life of these patients of the phase I study. With regard to adequate patient information, consent on a trial basis and expectations of therapy can provide a fully informed choice by the patient. Note that many, by volunteering, make a decision to stay hopeful in active treatment and also show altruism by collaborating in the development of new therapies [26].

## Conclusion

In conclusion, phase I studies have growing importance in the current scenario of clinical research as the starting position in the chain of clinical trials leading to the approval of new drugs. We believe that greater disclosure on the subject and the knowledge of the main characteristics of phase I studies may increase the referral of patients to research centres. Because many patients with advanced solid tumours have incurable disease, participation in a phase I study may be a therapeutic strategy that offers low risk and some chance of benefit.

## Declaration of conflict of interest

The authors have no conflicts of interest.

## Figures and Tables

**Table 1.** Concepts and terminologies employed in phase I studies.

English term	Suggested Portuguese term	Explanation
Dose limiting toxicities (DLT)	Toxicidade dose-limitante	Side effects deemed to be linked to the drug, unacceptable, limiting the increase of the dose.
Maximum tolerated dose (MTD)	Dose máxima tolerada (DTM)	Dose established from the occurrence of DLT in a given percentage of the study subjects; usually preconised from the occurrence of DLT in a third or more of the participants of an escalation stage.
Recommended phase II dose (RP2D)	Dose recomendada para estudo de fase II (DRF2)	Dose that should be used in subsequent phase II studies; usually established from the MTD (the MTD itself or a dose below the MTD) or of the optimum biological dose (OBD)
Optimum biologic dose (OBD)	Dose biológica ideal (DBI)	Dose linked to a pre-established effect on a biomarker, e.g. target inhibition or desired laboratorial parameter.
Dose lethal 10 (DL10)	Dose lethal para 10% (DL10)	Dose able to kill 10% of participants.
Dose escalation cohort	Coorte de escalonamento de dose	Initial cohorts of a phase I study in which rising doses of a drug under evaluation are administered to patients in order to determine the MTD and/or RP2D
Dose expansion cohort	Coorte de expansão de dose	Cohorts of a phase I study with a fixed agent dose to best assess any relevant aspect (toxicity, pharmacodynamics, pharmacokinetics or clinical activity)

**Table 2.** Main phase I study designs.

Design	Description
3 + 3	Cohorts of three patients received escalating doses – where one is DLT, include three more; the DRF2 is the dose corresponding to the previous stage in which 33% of patients had DLT.
Accelerated titration	Using a model for dose-toxicity monitoring providing faster initial scheduling, and the scheduling and descaling for the same patient.
Pharmacologically guided dose escalation	Model variation 3 + 3 using pharmacokinetics analysis pharmacologically guided or allowing for rapid dose escalation until achieving the established target concentration in the preclinical study DLT, then following with smaller increases.
Rotation of six	Model guided by rule where between two and six patients are allowed per stage, increasing the scaling speed and decreasing the exposure to subdoses with fewer patients in the early stages.
Continuous reassessment	Treatment of cohorts with initial dose close to MTD (based on preclinical data) and performance of DLT estimated based on data from each new patient entering each dose stage allowing scaling and descaling.
Scheduling with overdose control	Similar to the continuous reassessment, however, to evaluate probability to exceed the MTD for each step of the dose-interruption indication from a certain critical pre-specified probability value.
Time to event	Incorporates the evaluation of toxicity and efficacy for determining acceptable dose based on the odds of treatment efficacy (using standard response criteria or surrogate endpoints) and toxicity.

DLT : Dose-limiting toxicity;

DRF2: recommended dose for phase II study;

MTD: maximum tolerated dose.

## References

[1] Umscheid CA, Margolis DJ, Grossman CE (2011). Key concepts of clinical trials: a narrative review. Postgrad Med.

[2] Chabner BA (2011). Early approval for highly targeted cancer Drugs. N Engl J Med.

[3] LoRusso PM, Boerner SA, Seymour L (2010). An overview of the optimal planning, design, and conduct of phase I studies of new therapeutics. Clin Cancer Res.

[4] Internet: Agência Nacional de Vigilância Sanitária and Ministério da Saúde. Boas Práticas Clínicas (2005). Documento das Américas. IV Conferência Pan-Americana para Harmonização da Regulamentação Farmacêutica. República Dominicana.

[5] Ivy SP, Siu LL, Garret-Mayer E (2010). Approaches do phase I clinical trial design focused on safety, efficiency, and selected patient populations: a report from the clinical trial design task force of the national cancer institute investigational drug steering committee. Clin Cancer Res.

[6] Sleijfer S, Wiemer E (2008). Dose selection in phase I studies: why we should always go for the top. J Clin Oncol.

[7] Mandrekar SJ, Qin R, Sargent DJ (2010). Model-based phase I designs incorporating toxicity and efficacy for single and dual agent drug combinations: Methods and challenges. Stat Med.

[8] Booth CM (2008). On behalf of the task force on methodology for the development of innovative cancer therapies. endpoints and other considerations in phase I studies of targeted anticancer therapy: recommendations from the task force on Methodology for the Development of Innovative Cancer Therapies (MDICT). Eur J Cancer.

[9] Tolcher AW, Takimoto CH, Rowinsky EK (2002). The multifunctional, multi-institutional, and sometimes even global phase I study: a better life for phase I evaluations or just “living large”?. J Clin Oncol.

[10] Skolnik JM (2008). Shortening the timeline of pediatric phase I trials: the rolling six design. J Clin Oncol.

[11] Le Tourneau C, Lee JJ, Siu LL (2009). Dose escalation methods in phase I cancer clinical trials. J Natl Cancer Inst.

[12] Iasonos A, O’Quigley J (2014). Adaptive dose-finding studies: a review of model-guided phase I clinical trials. J Clin Oncol.

[13] Manji A (2013). Evolution of clinical trial design in early drug development: systematic review of expansion cohort use in single-agent phase I cancer trials. J Clin Oncol.

[14] Hyman DM (2014). Nomogram to predict cycle-one serious drug-related toxicity in phase I oncology trials. J Clin Oncol.

[15] Bedard PL, Siu LL (2014). Tilting the balance of dose modification for oral anticancer drugs?. J Clin Oncol.

[16] Postel-Vinay S (2011). Phase I trials of molecularly targeted agents: should we pay more attention to late toxicities?. J Clin Oncol.

[17] Wagesa NA, Conawayb MR, O’Quigley J (2011). Dose-finding design for multi-drug combinations. Clin Trials.

[18] Horstmann E (2005). Risks and benefits of phase 1 oncology trials, 1991 through 2002. N Engl J Med.

[19] Jardim DLF (2014). Predictive value of phase I trials for safety in later trials and final approved dose: analysis of 61 approved cancer drugs. Clin Cancer Res.

[20] Tsimberidou AM (2012). Personalized medicine in phase I clinical trials program: the MD Anderson Cancer Center initiative. Clin Cancer Res.

[21] Jain RK (2010). Phase I oncology studies: evidence that in the era of targeted therapies patients on lower doses do not fare worse. Clin Cancer Res.

[22] Ryan CJ (2010). Phase I clinical trial of the CYP17 inhibitor abiraterone acetate demonstrating clinical activity in patients with castration-resistant prostate cancer who received prior ketoconazole therapy. J Clin Oncol.

[23] Tai WMD (2013). Do elderly patients benefit from enrollment in phase I clinical trials?. J Clin Oncol.

[24] Schwandt A (2014). The role of age on dose-limiting toxicities in phase I dose-escalation trials. Clin Cancer Res.

[25] Henderson GE (2007). Clinical trials and medical care: defining the therapeutic misconception. PLoS Med.

[26] Agrawal M, Emanuel EJ (2003). Ethics of phase 1 oncology studies: reexamining the arguments and data. JAMA.

